# Growth and lipid accumulation of microalgae from fluctuating brackish and sea water locations in South East Queensland—Australia

**DOI:** 10.3389/fpls.2015.00359

**Published:** 2015-05-19

**Authors:** Van Thang Duong, Skye R. Thomas-Hall, Peer M. Schenk

**Affiliations:** Algae Biotechnology Laboratory, School of Agriculture and Food Sciences, The University of QueenslandBrisbane, QLD, Australia

**Keywords:** biodiesel, diatom, fatty acids, microalgae, omega-3 fatty acids

## Abstract

One challenge constraining the use of microalgae in the food and biofuels industry is growth and lipid accumulation. Microalgae with high growth characteristics are more likely to originate from the local environment. However, to be commercially effective, in addition to high growth microalgae must also have high lipid productivities and contain the desired fatty acids for their intended use. We isolated microalgae from intertidal locations in South East Queensland, Australia with adverse or fluctuating conditions, as these may harbor more opportunistic strains with high lipid accumulation potential. Screening was based on a standard protocol using growth rate and lipid accumulation as well as prioritizing fatty acid profiles suitable for biodiesel or nutraceuticals. Using these criteria, an initial selection of over 50 local microalgae strains from brackish and sea water was reduced to 16 strains considered suitable for further investigation. Among these 16 strains, the ones most likely to be effective for biodiesel feedstock were *Nitzschia* sp. CP3a, *Tetraselmis* sp. M8, *Cymbella* sp. CP2b, and *Cylindrotheca closterium* SI1c, reaching growth rates of up to 0.53 day^−1^ and lipid productivities of 5.62 μg mL^−1^day^−1^. Omega-3 fatty acids were found in some strains such as *Nitzschia* sp. CP2a, *Nitzschia* sp. CP3a and *Cylindrotheca closterium* SI1c. These strains have potential for further research as commercial food supplements.

## Introduction

Microalgae grow in most natural environments, typically aquatic and marine systems, but they are also found in soil, ice, rock pools or in volcanic water that can have extreme environmental fluctuations (Duong et al., 2012). Microalgae suffering from adverse or fluctuating conditions often have the ability to accumulate higher contents of biochemical products for survival, such as lipid, starch, protein or carotenoid contents (Lim et al., 2012). Microalgae can produce a variety of lipids that can be used by the food and biofuel industry (Huerlimann et al., 2010). For the production of biodiesel, fatty acids with a chain length of 14–18 carbons are preferable. Saturated fatty acids C14, C16, and C18 and unsaturated fatty acids such as C16:1, C16:2, C18:1, and C18:2 are the most important for producing good biodiesel quality (Schenk et al., 2008). This is because the other unsaturated fatty acids with 3 or 4 double bonds have reduced stability in storage (Knothe, 2006; Chisti, 2007).

The components of fatty acids, including saturated and unsaturated fatty acids produced by microalgae, differ for different species and strains (Renaud et al., 1994; Salama et al., 2013). The accumulation of fatty acid components can be controlled by environmental conditions such as temperature, nutrient availability and salinity (Renaud et al., 2002; Huerlimann et al., 2010). Alternatively, the necessary fatty acids can be developed using a chemical process. For example, unsaturated fatty acids from microalgae, especially fatty acids with 3 or 4 double-bonds, can be easily hydrogenated under partial catalysts (Jang et al., 2005), but it is preferable to identify and propagate microalgae that do not require secondary treatment to modify their fatty acid profile. Growth temperature is a major factor that dramatically influences lipid accumulation in general or lipid composition in particular. For instance, a higher growth temperature can stimulate lipid accumulation in microalgae or a lower growth temperature can lead to increased saturated fatty acid composition (Renaud et al., 2002).

The screening of microalgae is not limited to those suitable for biodiesel production, because microalgae also offer multiple bio-products that can be used in a variety of industry (Schenk et al., 2008; Huerlimann et al., 2010). For instance, unsaturated fatty acids containing more than three double bonds, including omega-3 fatty acids, are not preferable for biodiesel but they can be useful as nutritious food or feed supplement. These compounds currently have a high value and could compete with fish oil in terms of productivity and environmental sustainability (Adarme-Vega et al., 2014).

Although microalgae are known to produce considerable amounts of lipids this it is not true for all microalgal strains. The purpose of our research was to screen microalgal isolates collected from brackish and sea water and identify those with the highest growth and lipid production with desirable fatty acid composition. As lipid accumulation capacity of algae changes when subjected to adverse external factors, such as nutrient supply and environmental conditions (Huerlimann et al., 2010; Chen et al., 2011), the research focused on microalgae from sites that have known adverse or fluctuating environmental conditions (e.g., intertidal rock pools; Underwood, 1984). After isolating pure algal cultures, growth and lipid production were compared to establish which strains are most stable and productive and most likely to be effective as a feedstock source for biofuel production. In addition, strains which produce fatty acids suitable for the health food industry were identified.

## Materials and methods

Microalgae samples were collected from a variety of sites in the lower reaches of the Brisbane River, Stradbroke Island, and Sunshine Coast in South East Queensland—Australia. The samples represent different environmental conditions of tidal brackish river water (50 cm depth), rock pools and beaches. Two species from the Australian National Algae Culture Collection from the Commonwealth Scientific and Industrial Research Organisation (CSIRO) were used to compare growth and lipid production with the strains that were isolated from the field (Table 1). The microalgae were intentionally gathered from different environments to provide a diverse taxonomy, stored in a cold box and transferred to the laboratory for analysis.

**Table 1:** **Sources and accessions of microalgae used in this study**.

**Species**	**GPS coordinates**	**Species origin and collection time**	**Site status**	**Genbank accession number**
*Achnanthes* sp. BR22.5_3	27°29′29.00S 153°00′48.00E	Brisbane River (11.40 am 22/05/2012)	Brackish water	KF 360813
*Bacillariophyta* sp. B3	26°48′12.11S 153°08′50.86E	Bullcock Beach (3 pm 12/06/2011)	Tidal rock pool	KF 360815
*Bacillariophyta* sp. SI1a	27°26′14.23S 153°30′51.08E	Stradbroke Island (3 pm 07/07/2011)	Brackish rook pool	KF 360816
*Chlorella* sp. BR2	–	Brisbane River Lim et al., 2012	Brackish water	–
*Cylindrotheca closterium* SI1c	27°26′14.23S 153°30′51.08E	Stradbroke Island (3 pm 07/07/2011)	Brackish rock pool	KF 360818
*Cymbella cistuliformis* CP2c	24°59′19.96S 153°21′04.74E	Frazer Island, Champagne Pools (10 am 1/4/2012)	Tidal rock pool	KF 360819
*Cymbella* sp. CP2b	24°59′19.96S 153°21′04.74E	Frazer Island, Champagne Pools (10 am 1/4/2012)	Tidal rock pool	KF 360820
*Dunaliella tertiolecta*	–	CSIRO Tasmania (CS-175/8)	–	–
*Navicula* sp. BR22.52	27°29′29.00S 153°00′48.00E	Brisbane River (11.40 am 22/05/2012)	Brackish water	KF 360822
*Navicula* sp. CP6a	24°59′19.96S 153°21′04.74E	Frazer Island, Champagne Pools (10 am 1/4/2012)	Tidal rock pool	KF 360823
*Navicula* sp. SI2d	27°25′33.82S 153°31′45.70E	Stradbroke Island (3 pm 07/07/2011)	Tidal rock pool	KF 360824
*Nitzschia* sp. CP2a	24°59′19.96S 153°21′04.74E	Frazer Island, Champagne Pools (10 am 1/4/2012)	Tidal rock pool	KF 360825
*Nitzschia* sp. CP3a	24°59′19.96S 153°21′04.74E	Frazer Island, Champagne Pools (10 am 1/4/2012)	Tidal rock pool	KF 360826
*Phaeodactylum tricornutum*	–	CSIRO Tasmania (CS-29/8)	–	–
*Tetraselmis* sp. M8	–	Rock Pool Maroochydore, Lim et al., 2012	Tidal rock pool	JQ 423158
*Thalassiosira rotula* SI2a	27°25′33.82S 153°31′45.70E	Stradbroke Island (3 pm 07/07/2011)	Tidal rock pool	KF 360828

### Isolation and preliminary screening

Microalgae were cultured in f/2 medium at 25 ± 1°C with a 12/12 h light/dark photoperiod at a light intensity of 120 μmol photons m^−2^s^−1^ from fluorescent lights (Osram L36W/840) and constant bubbling conditions (LP-100 air pump, Shen-Zhen Xingrisheng Industrial Co. Ltd) in a temperature-controlled environment room. Single cells were isolated by a micropipette on an inverted microscope and grown in 96 wells plates before transferring to 100 mL flasks for pure cultivation as described previously (Duong et al., 2012). The isolation procedure was conducted with sterilized equipment and in the laminar flow. Single cells were isolated by the micromanipulation method and the pure culture was checked regularly for the presence of contaminating algae or high abundance of bacteria during inoculation and growth experiments. It should be mentioned that the cultures in this study are not axenic. While it can be excluded that they contain other microalgae or protists, they still contain associated bacteria. This was considered desirable as microalgae-associated bacteria often provide improved growth (Amin et al., 2012). Algal strains were then provisionally screened based on their ability for rapid growth and lipid fluorescence using Nile red staining as described by Lim et al. (2012).

### Classification by DNA sequencing

Microalgal biomass was collected at the late exponential phase of cultivation for DNA extraction. DNA extraction was performed using a phenol:chloroform method. After extraction, genomic DNA within the 18S rRNA region was amplified on a PCR machine by using the following primers: Forward 5′-GCGGTAATTCCAGCTCCAATAGC-3′ and Reverse 5′-GACCATACTCCCCCCGGAACC-3′. The process followed was developed by Lim et al. (2012). PCR templates were then purified by using a Wizard SV Gel PCR Clean-Up System (Promega). For sequencing preparation, 5 μL of a 25 ng μL^−1^ PCR product were combined with 1 μL of a 10 μM solution of each of the above primers. The reaction was topped up to 12 μL with Millipore water in a 1.5 mL tube and sent to the Australian Genome Research Facility (AGRF) at The University of Queensland for sequencing and analysis. The DNA sequence data was compared with Genbank entries for classification.

### Standard protocol for growth experiments

After obtaining pure cultures, all isolated strains were grown in f/2 medium following a cultivation protocol that used bubbling for aeration and mixing. The standard protocol can be briefly described as follows: All strains were inoculated with 5 mL from a recently grown master culture and cultured in 150 mL f/2 medium until the end of the exponential growth phase was reached (less than 10% cell density increase/day) before starting the standard growth experiment. This culture was then used as the inoculum at a ratio of 1/10 for 300 mL f/2 medium in 400 mL bottles that were connected to a bubbling system and exposed to 12/12 h light/dark photoperiod at a light intensity of 120 μmol photons m^−2^s^−1^. Cells were counted daily by using a hemocytometer. Nitrate concentrations were monitored daily until the nutrient levels reached zero (below detection). Nitrate was determined by using a colorimetric assay (API test kit) and measured on a spectrophotometer at a wavelength 545 nm. Growth rates were calculated by the following equation (Levasseur et al., 1993)

$$K^{\prime }=\frac{\begin{array}{c} \;\\ ln\frac{N_{2}}{N_{1}} \end{array}}{t_{2}-t_{1}}$$

where N_1_ and N_2_ equal cell counts at time 1 (t_1_) and time 2 (t_2_), respectively. Doubling time was also calculated once the specific growth rate was known.

$$Doubling\;time=\;\frac{ln2}{K^{\prime }}$$

Microalgae were cultured for another 3 days after the nitrate concentration in the medium became undetectable to induce lipid biosynthesis.

### Fatty acid methyl esters (FAME) analysis

Samples for FAME analysis were collected when lipid accumulation reached its peak, after 3 days of starvation. A total of 4 mL of microalgal culture was collected and centrifuged at 8000 × g for 5 min. Biomass was collected and dried by a vacuum pump for 30 min. Lipids in the microalgal pellet were hydrolyzed and methyl-esterified in 300 μL of a 2% H_2_SO_4_ and methanol solution for 2 h at 80°C. Prior to the reaction, 50 μg of heneicosanoic acid (Sigma, USA) was added as internal standard. After the esterification step, 300 μL of 0.9% (w/v) NaCl solution and 300 μL of hexane were added and mixed for 20 s. To separate the phase, samples were centrifuged at 16,000 × g for 3 min. A total of 1 μL of hexane layer was injected into an Agilent 6890 gas chromatograph (GC) coupled to a 5975 MSD mass spectrometer (MS). The running conditions were described by Agilent's RTL DBWax method (Brown, 1991).

## Results

### Growth of selected microalgal strains

**Figure 1:**
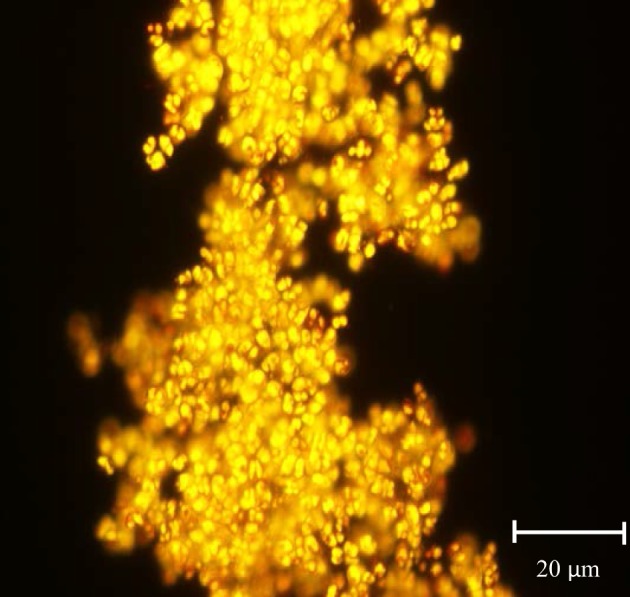
***Cymbella*** **sp. CP2b after Nile red staining under fluorescence microscopy**. Yellow droplets show lipid containing triacylglycerides and orange droplets show autofluorescence from chlorophyll. *Cymbella* sp. CP2b displayed fast growth rates and one of the highest lipid productivities (4.51 μg mL^−1^ day^−1^).

Out of 50 microalgal strains that were isolated, 16 were shortlisted based on their rapid growth and high lipid fluorescence following Nile red staining (Figure 1 shows an example). Strains were then compared in standard growth and lipid accumulation assays to determine the most suitable strains as feedstock for biodiesel and/or nutraceuticals. The growth of microalgal strains in the collection is presented in two groups for easier comparison: diatoms and green microalgae. The fastest growing strain *Thalassiosira rotula* SI2a grew 5.3 times faster than the slowest growing strain *Bacillariophyta* sp. B3 (Table 2). Among the diatomaceous species, *Thalassiosira rotula* SI2a's cell density was 2.47 × 10^6^ cells mL^−1^ and this strain exhibited the highest growth rate and lowest doubling time, achieving 0.64 day^−1^ and 1.09 days, respectively. The cell density of *Phaeodactylum tricornutum* reached 6.97 × 10^6^ cells mL^−1^ and grew more after the standard assay's completion. The growth rate and doubling time of this strain was 0.49 day^−1^ and 1.42 days, respectively. *Cylindrotheca closterium* SI1c's growth reached a peak of 1.89 × 10^6^ cells mL^−1^. *Bacillariophyta* sp. SI1a, *Bacillariophyta* sp. B3, and *Navicula* sp. SI2d grew slowly and reached peaks of 3.18 × 10^5^ cells mL^−1^, 4.5 x 10^4^ cells mL^−1^ and 1.18 × 10^5^ cells mL^−1^, respectively.

**Table 2:** **Growth characteristics of the isolated strains in f/2 medium during the duration of the standard assay**.

**Species**	**Mean growth rate (μ day^−1^)**	**Doubling time (days)**	**Maximum cell density (x10^4^ cells mL^−1^)**
*Achnanthes* sp. BR22.5_3	0.13	5.48	28.50
*Bacillariophyta* sp. B3	0.12	5.96	6.17
*Bacillariophyta* sp. SI1a	0.32	2.16	31.83
*Chlorella* sp. BR2	0.45	1.54	245.83
*Cylindrotheca closterium* SI1c	0.53	1.31	189.00
*Cymbella cistuliformis* CP2c	0.26	2.65	28.67
*Cymbella* sp. CP2b	0.53	1.31	103.17
*Dunaliella tertiolecta*	0.46	1.51	266.83
*Navicula* sp. BR22.52	0.34	2.05	24.50
*Navicula* sp. CP6a	0.39	1.80	42.33
*Navicula* sp. SI2d	0.29	2.40	11.83
*Nitzschia* sp. CP2a	0.22	3.08	298.33
*Nitzschia* sp. CP3a	0.17	3.99	123.83
*Phaeodactylum tricornutum*	0.49	1.42	696.67
*Tetraselmis* sp. M8	0.43	1.60	262.17
*Thalassiosira rotula* SI2a	0.64	1.09	247.00

*Shown are mean values from three separately-grown cultures each*.

*Dunaliella tertiolecta, Chlorella* sp. BR2 and *Tetraselmis* sp. M8 were the fastest growing green algal strains. Cell density of three strains increased gradually and peaked after 7 to 9 days. The cell density peaks for *Chlorella* sp. BR2, *Tetraselmis* sp. M8, and *Dunaliella tertiolecta* were 2.46 × 10^6^ cells mL^−1^, 2.62 × 10^6^ cells mL^−1^, and 2.67 × 10^6^ cells mL^−1^, respectively. These strains had similar growth rates and doubling times (Table 2).

**Figure 2:**
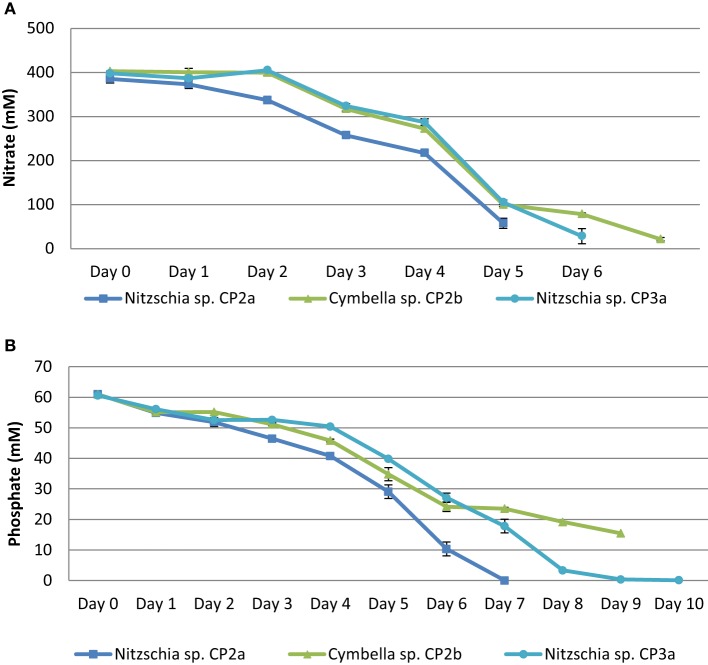
**Nitrate (A) and phosphate (B) depletion in growth medium (daily uptake) of** ***Cymbella*** **sp. CP2b**, ***Nitzschia*** **sp. CP2a, and** ***Nitzschia*** **sp. CP3a**. Shown are mean values ± SEs from three separately-grown cultures each.

Nitrogen and phosphorous concentrations changed during the cultivation of the strains. Nitrate concentration in the medium decreased dramatically from day 3 to day 5 and nitrogen depletion occurred from day 5 to day 7 of the experiment. The concentration of phosphate decreased gradually over the experimental period. A depletion of phosphate occurred from day 7 to day 10. *Nitzschia* sp. CP2a and *Nitzschia* sp. CP3a consumed most nutrients among the strains tested (Figure 2).

### Lipid contents in microalgae collection

**Figure 3:**
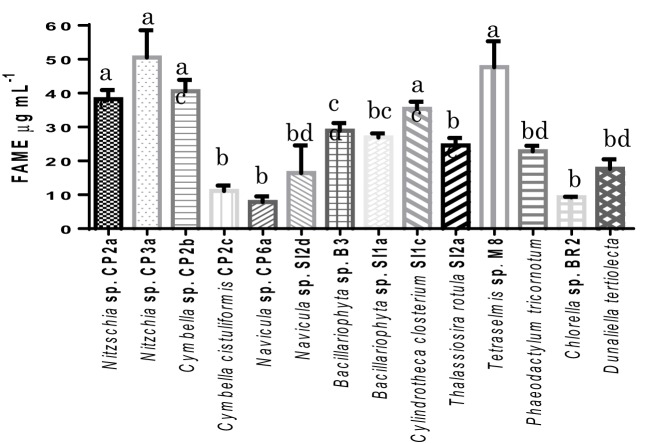
**Total fatty acid methyl esters (FAMEs) contents of the isolated microalgal strains, expressed in μg mL^−1^**. Shown are mean values ± SEs from three separately-grown cultures. Different letters above bars indicate statistically significant differences (*p* < 0.05; Two-Way ANOVA, Tukey's HSD test using GraphPad Prism 6.0).

The result from GC/MS showed that all selected microalgal strains can produce fatty acids. Eighteen different fatty acids were detected, however not all strains produced all 18 fatty acids. There was no detection of some unsaturated fatty acid with 3 or 4 double bonds such as hexadecatrienoic acid, hexadecatetraenoic acid, and stearidonic acid in *Nitzschia* sp. CP2a, *Cymbella* sp. CP2b, *Cymbella cistuliformis* CP2c, *Nitzschia* sp. CP3a, and *Navicula* sp. CP6a. Total FAMEs ranged from 9.27 to 50.53 μg mL^−1^. The highest FAME content was achieved for *Nitzchia* sp. CP3a and the lowest was measured for *Chlorella* sp. BR2 (Figure 3). Palmitic acid and stearic acid were dominant among the total fatty acids. The percentage of palmitic acid ranged from 19.83 to 37.65% in most of the strains, except for *Bacillariophyta* sp. SI1a, *Cylindrotheca closterium* SI1c, *Thalassiosira rotula* SI2a, and *Navicula* sp. SI2d. Stearic acid that accumulated in the strains ranged from 14.81 to 57.73%, except for *Nitzschia* sp. CP2a, *Cymbella* sp. CP2b, and *Nitzschia* sp. CP3a.

Interestingly, omega-3 eicosapentaenoic acid (EPA) and docosahexaenoic acid (DHA) were found in most of the strains. The highest EPA percentage of total FAMEs reached 30.85% for *Phaeodactylum tricornotum*, 14.49% for *Nitzschia* sp. CP3a, and 12.46% for *Nitzschia* sp. CP2a. DHA reached 1.11 and 1.36% for *Nitzschia* sp. CP3a and *Nitzschia* sp. CP2a, respectively. On average, saturated fatty acids were the major FAMEs in most strains; the highest values were found in *Thalassiosira rotula* SI2a (62.18%), *Bacillariophyta* sp. B3 (71.9%) and *Navicula* sp. CP6a (71.61%). However, total unsaturated fatty acids were higher than total saturated fatty acids in strains such as *Dunaliella tertiolecta* (62.08%), *Cylindrotheca closterium* SI1c (61.65%), and *Nitzschia* sp. CP3a (60.47%).

## Discussion

All strains in the study were collected from estuary, coastal waters and rock pools where environmental conditions change frequently, especially changes of temperature, salinity, light exposure, and nutrients. The changes require microalgae to adapt and/or produce more biochemical products to ensure their survival (Lim et al., 2012). Lipid is one of the biochemical products that microalgae can produce along with starch under nutrient deprived conditions in the light (Schenk et al., 2008). Lipid productivities were highest for *Nitzschia* sp. CP3a (5.62 μg mL^−1^ day^−1^)*, Tetraselmis* sp. M8 (5.29 μg mL^−1^ day^−1^)*, Cymbella* sp. CP2b (4.51 μg mL^−1^ day^−1^), and *Cylindrotheca closterium* SI1c (3.93 μg mL^−1^ day^−1^). The value for *Tetraselmis* sp. M8 was higher than the lipid productivity previously reported for this strain when grown under laboratory conditions (2.1 μg mL^−1^ day^−1^; Lim et al., 2012). It is interesting to note that all of these strains have been isolated from coastal rock pools subjected to tides. Their high lipid productivities suggest that these microalgae may be more opportunistic than others from more stable environments (e.g., stationary lakes or open ocean). A future study comparing the growth rates and lipid productivities of microalgae isolated from stable and unstable environments may further substantiate this notion. High growth rates of these microalgae lead to rapid production of biomass while their high lipid accumulation capability is likely to assist in higher survival rates during adverse conditions. Interestingly, divergent from this trend is *Nitzschia* sp. CP3a which despite its high lipid productivity had a very slow growth rate. Further studies should be conducted to test the survival rates of these strains and if these correlate with their cellular lipid and/or starch contents.

Diatoms can survive in harsh environments (Seckbach, 2007) and usually produce more lipids in harsher environments (Lim et al., 2012). For this research, the difference of environmental conditions between natural habitats and the laboratory may have created a harsh environment that impacted on growth and chemical accumulation of microalgae tested. Many of the strains in this study adapted to their new environment and grew well under laboratory conditions. For instance, *Cymbella* sp. is a diatom that usually requires multi nutrients and minerals from natural sources (Tarapchak et al., 1983; Takeda, 1998). However, this strain grew well in f/2 medium and displayed one of the best growth rates (Table 2). The indoor growth experiments were set up for conditions similar to the natural habitats (temperature, nutrients, light). However, the indoor conditions are not identical to the natural conditions and the adaptability of the selected microalgae to the laboratory condition is a useful indicator of microalgae's ability to adapt to different external conditions which also will be encountered when grown at large scale for commercial purposes.

In addition, nutrient availability also affects diatom growth. For example, silica is the main components of the diatom cell wall. Changing nutrient availability may directly influence the structure of silica composition in the cell wall and affect diatom growth (Tarapchak et al., 1983). Thus optimizing nutrient availability can also substantially affect growth rates and biomass production. For example, increasing of nutrients or nitrogen leads to an increase of growth rate (Converti et al., 2009; Chen et al., 2011; Borowitzka and Moheimani, 2013). However, to enable a direct side-by-side comparison, a standard assay with unoptimized parameters was used in the current study. Clearly, higher growth rates and lipid productivities are achievable after careful cultivation optimization. For example, this has been achieved for *Tetraselmis* sp. M8 (Sharma et al., 2014). A comparison of growth rates among the fastest growth strains between the study and other publications has been undertaken. It showed that the growth of the strains in the present study was similar or faster to those in other studies for *Chlorella* sp. BR2, *Tetraselmis* sp. M8 (24.5 and 18.6% increase, respectively, compared to the study by Lim et al., 2012) or slightly lower for *Thalassiosira rotula* (23.4% reduction compared to the study by Doan et al., 2011). The difference may be due to a difference of strains used and growth conditions, such as air and nutrient supply, temperature, culture systems and photosynthesis periods (Renaud et al., 2002; Converti et al., 2009).

Total FAMEs results from GC/MS analysis showed that all 14 microalgal strains tested were lipid producers under the standard conditions used (Table 3). *Nitzschia* sp. CP3a was the highest lipid producer, followed by *Tetraselmis* sp. M8, *Cymbella* sp. CP2b, *Nitzschia* sp. CP2a and *Cylindrotheca closterium* SI1c. The lipid content produced by *Tetraselmis* sp. M8 was similar to the results of Lim et al. (2012). The proportion of saturated fatty acids reached more than 50% for most of the diatom strains and was dominated by myristic acid, palmitic acid, and stearic acid. These fatty acids are the main desirable components for biodiesel production (Schenk et al., 2008). Based on the above properties, we conclude that the two diatoms, *Cymbella* sp. CP2b and *Cylindrotheca closterium* SI1c, are potential strains that can be used to develop microalgal biofuel production.

**Table 3:** **Fatty acid composition in percentage of total fatty acid methyl esters (FAMEs) of different microalgal strains collected in South East Queensland, Australia after cultivation to nitrate depletion and an additional 3 days of starvation**.

**Fatty acids**	***Bacillariophyta* sp. *B3***	***Bacillariophyta* sp. *SI1a***	***Cylindrotheca* closterium SI1c**	***Thalassiosira* rotula SI2a**	***Navicula* sp. *SI2d***	***Tetraselmis* sp. *M8***	***Phaeodactylum* tricornotum**	***Chlorella* sp. *BR2***	***Dunaliella* tertiolecta**	***Nitzschia* sp. *CP2a***	***Cymbella* sp. *CP2b***	***Cymbella* cistuliformis CP2c**	***Nitzschia* sp. *CP3a***	***Navicula* sp. *CP6a***
Lauric (C12:0)	1.12	1.64	1.59	2.17	2.18	0.95	0.13	2.05	0.27	0.00	0.00	0.00	0.00	0.00
Myristic (C14:0)	21.32	0.74	0.63	0.66	0.81	0.93	8.74	1.65	0.47	5.96	9.56	6.73	5.33	4.88
Palmitic (C16:0)	23.64	0.88	0.23	1.27	1.12	28.33	29.31	31.68	19.87	30.10	37.65	30.22	28.82	37.04
Palmitoleic (C16:1)	0.45	1.43	4.66	1.19	1.37	1.79	0.21	4.71	0.35	31.57	38.26	21.31	27.28	15.75
Hexadecadienoic (C16:2)	5.08	13.72	8.97	3.18	6.56	14.59	0.43	1.42	1.44	2.80	1.60	2.49	3.33	1.63
Hexadecatrienoic (C16:3)	4.80	23.29	23.28	2.32	6.92	18.27	5.59	8.84	4.22	0.00	0.00	0.00	0.00	0.00
Hexadecatetraenoic (C16:4)	0.72	1.85	1.77	0.93	2.06	0.25	0.01	0.04	23.30	0.00	0.00	0.00	0.00	0.00
Stearic (C18:0)	25.15	39.13	35.21	57.73	52.32	19.79	14.81	29.28	16.65	5.71	6.56	20.39	5.25	28.74
Oleic (C18:1)	3.57	5.57	10.11	19.24	18.07	8.08	7.13	11.86	0.82	1.97	1.18	2.37	2.92	2.07
Linoleic (C18:2)	1.48	0.21	0.17	0.09	0.05	0.24	1.76	6.12	1.85	0.80	0.28	1.21	1.33	0.38
Linolenic (C18:3)	5.64	6.90	10.83	9.95	4.23	3.41	0.34	0.39	29.98	1.79	0.09	0.61	1.16	0.26
Stearidonic (C18:4)	0.10	0.15	0.20	0.10	0.15	0.14	0.00	0.13	0.00	0.00	0.00	0.00	0.00	0.00
Arachidic (C20:0)	0.52	0.18	0.42	0.17	0.11	0.26	0.60	1.28	0.57	0.19	0.20	0.77	0.13	0.95
Arachidonic (C20:4)	0.12	3.20	0.67	0.04	0.13	1.54	0.00	0.04	0.00	5.28	0.68	4.80	8.85	1.87
Eicosapentaenoic (EPA) (C20:5)	5.71	0.28	0.38	0.21	3.44	0.28	30.85	0.08	0.12	12.46	3.94	8.75	14.49	6.16
Behenic (C22:0)	0.15	0.40	0.27	0.18	0.19	0.34	0.00	0.00	0.00	0.00	0.00	0.00	0.00	0.00
Docosatetraenoic (C22:4)	0.26	0.25	0.29	0.32	0.18	0.44	0.00	0.00	0.00	0.00	0.00	0.00	0.00	0.00
Docosahexaenoic (DHA)(C22:6)	0.17	0.17	0.34	0.26	0.09	0.36	0.00	0.00	0.01	1.36	0.00	0.34	1.10	0.28
Saturated fatty acids (%)	71.90	42.96	38.35	62.18	56.73	50.60	53.59	65.93	37.82	41.96	53.97	58.11	39.53	71.61
Unsaturated fatty acids (%)	28.10	57.04	61.65	37.82	43.27	49.40	46.31	33.64	62.08	58.04	46.03	41.89	60.47	28.39
Total fatty acids (μg mL^−1^)	28.91	26.91	35.36	24.56	16.41	47.65	22.85	9.27	17.69	38.21	40.58	11.13	50.53	7.90
Lipid productivity (μg mL^−1^ day^−1^)	3.21	2.99	3.93	2.73	1.82	5.29	2.54	1.03	1.97	4.25	4.51	1.24	5.62	0.88

*Shown are mean values from three separately-grown cultures each*.

## Author contributions

All authors designed the work, wrote the manuscript and approve the final version of the manuscript. VD acquired the material. VD, ST carried out the experiments, analyzed and interpreted the data. ST, PS critically revised the work and the manuscript.

### Conflict of interest statement

The authors declare that the research was conducted in the absence of any commercial or financial relationships that could be construed as a potential conflict of interest.
